# Autonomic responses to cold face stimulation in sickle cell disease: a time-varying model analysis

**DOI:** 10.14814/phy2.12463

**Published:** 2015-07-14

**Authors:** Patjanaporn Chalacheva, Roberta M Kato, Suvimol Sangkatumvong, Jon Detterich, Adam Bush, John C Wood, Herbert Meiselman, Thomas D Coates, Michael C K Khoo

**Affiliations:** 1 Department of Biomedical Engineering, Viterbi School of Engineering, University of Southern CaliforniaLos Angeles, California, USA; 2 Department of Pediatrics, Children’s Hospital of Los AngelesLos Angeles, California, USA; 3 Department of Physiology and Biophysics, Keck School of Medicine, University of Southern CaliforniaLos Angeles, California, USA

**Keywords:** Autonomic nervous system, cold face stimulation, mathematical model, sickle cell disease

## Abstract

Sickle cell disease (SCD) is characterized by sudden onset of painful vaso-occlusive crises (VOC), which occur on top of the underlying chronic blood disorder. The mechanisms that trigger VOC remain elusive, but recent work suggests that autonomic dysfunction may be an important predisposing factor. Heart-rate variability has been employed in previous studies, but the derived indices have provided only limited univariate information about autonomic cardiovascular control in SCD. To circumvent this limitation, a time-varying modeling approach was applied to investigate the functional mechanisms relating blood pressure (BP) and respiration to heart rate and peripheral vascular resistance in healthy controls, untreated SCD subjects and SCD subjects undergoing chronic transfusion therapy. Measurements of respiration, heart rate, continuous noninvasive BP and peripheral vascular resistance were made before, during and after the application of cold face stimulation (CFS), which perturbs both the parasympathetic and sympathetic nervous systems. Cardiac baroreflex sensitivity estimated from the model was found to be impaired in nontransfused SCD subjects, but partially restored in SCD subjects undergoing transfusion therapy. Respiratory-cardiac coupling gain was decreased in SCD and remained unchanged by chronic transfusion. These results are consistent with autonomic dysfunction in the form of impaired parasympathetic control and sympathetic overactivity. As well, CFS led to a significant reduction in vascular resistance baroreflex sensitivity in the nontransfused SCD subjects but not in the other groups. This blunting of the baroreflex control of peripheral vascular resistance during elevated sympathetic drive could be a potential factor contributing to the triggering of VOC in SCD.

## Introduction

In sickle cell disease (SCD), red blood cells with sickle hemoglobin (HbS) undergo shape transformation and become rigid when HbS polymerizes, following the release of oxygen to the surrounding tissues (Rees et al. 2010). The delay between oxygen release and HbS polymerization is small (Eaton and Hofrichter 1995), and thus, if transit time of these red blood cells through the microvasculature is prolonged, sickling can lead to blood flow obstruction in some capillary beds. The clinical manifestation of extensive obstruction of microvascular flow is vaso-occlusive crisis (VOC), which is characterized by episodes of pain and subsequently organ damage or even more severe consequences. However, the mechanisms by which transient regional vaso-occlusion cascades into full-blown VOC remain unclear.

Recent studies from our laboratory and others point to significant sympathovagal imbalance of the autonomic nervous system (ANS) in SCD. Relative dominance of sympathetic drive promotes peripheral vasoconstriction and, as such, prolongs the transit time of sickle cells as they traverse the microvasculature. Thus, it is quite possible that autonomic dysfunction may be a key factor that predisposes SCD patients to VOC (Alexy et al. 2010; Sangkatumvong et al. 2011; Connes and Coates 2013). Previous studies have reported that decreased parasympathetic activity in SCD and sickle cell trait (SCT) subjects, and elevated cardiac sympathetic activity in SCT subjects (Pearson et al. 2005; Connes et al. 2006; Martins Wde et al. 2012). The association of greater parasympathetic withdrawal with disease severity has also been reported (Pearson et al. 2005). Sudden death, which is known to be associated with SCD, has been attributed to autonomic dysfunction (Romero Mestre et al. 1997). We have recently shown marked parasympathetic withdrawal in response to transient hypoxia (Sangkatumvong et al. 2008) in SCD subjects. As well, sighs are more frequently associated with drops in peripheral microvascular perfusion in SCD patients, making pulmonary-vasoconstriction coupling a critical and measurable trigger of VOC (Sangkatumvong et al. 2011).

Spectral analysis of heart-rate variability (HRV) has been employed as a ubiquitous tool for assessing ANS function due to the ease and accuracy with which heart rate can be monitored in humans. All previous studies of ANS function in SCD employed noninvasive measures derived from HRV. However, there are important limitations with this methodology that are frequently overlooked. Some prominent researchers have called into question the assumptions that underlie the use of spectral analysis for delineating the sympathetic from parasympathetic components of cardiac autonomic drive (Eckberg 1997; Malpas 2002). As well, changes or differences in breathing frequency, tidal volume, or ventilatory pattern can significantly confound the interpretation of autonomic activity that one derives from HRV spectral analysis (Brown et al. 1993). To circumvent the aforementioned limitations, in this study, we have supplemented conventional HRV spectral analysis with a model-based approach that allows us to quantify the strengths of the key functional mechanisms (such as baroreflex control of heart rate and respiratory-cardiac coupling) that contribute to HRV. This approach has also been applied to evaluate the relative contributions of the major functional mechanisms that account for fluctuations in peripheral resistance.

Since it is known that VOC can be triggered by cold (Resar and Oski 1991), we hypothesized that perturbing the ANS with cold face stimulation (CFS) would provide greater delineation between the responses in SCD subjects and those elicited in healthy controls. Cold face stimulation is a noninvasive test that activates both the cardiac parasympathetic and the peripheral sympathetic nervous system (Khurana et al. 1980). In normals, CFS generally leads to an initial bradycardia due to vagal stimulation, followed by a rise in BP due to sympathetically induced peripheral vasoconstriction. In this study, CFS was applied to healthy controls, SCD subjects participating in a chronic transfusion program, as well as SCD subjects not undergoing chronic transfusion. Subjects on chronic transfusion receive sufficient blood transfusion every 3 weeks to suppress hemoglobin S concentration below 30% and maintain a hemoglobin level above 9.5 g/dL. We hypothesized that the SCD subjects not participating in the transfusion program would exhibit altered autonomic function, but those in the chronic transfusion program would show partial recovery toward normal ANS status. To test these hypotheses, we incorporated the ability to accommodate time-varying changes in the parameters into the model, in order to take into account potential changes in the underlying dynamics resulting from transient exposure to the cold face stimulus.

## Methods

### Participants

All experiments were conducted at Children’s Hospital Los Angeles (CHLA). The study protocol was approved by the Committee on Clinical Investigations (institutional review board of CHLA). Participants were selected from healthy controls and SCD patients who were African American and 10 years or older. Some of the SCD subjects were participants in the chronic transfusion program. Those who recently had crisis or were hospitalized for sickle-related event (less than 2 weeks prior to the study), or unable to lay flat for 30 min or move from laying to standing posture were excluded. The participants were divided into three treatment groups: healthy controls (CTL), nontransfused SCD (NTF), and chronically transfused SCD (CTF). All but two SCD subjects were SS genotype (the two subjects were SB0 thalassemia and included in NTF group). There were (male/female) 4/5, 4/7 and 5/3 subjects in each respective group. The grouped average ages (mean ± standard error) were 25.7 ± 3.5, 23.1 ± 3.2 and 19.9 ± 3.7 years, respectively. Hemoglobin count in each group (CTL, NTF, and CTF) was 13.6 ± 0.91, 9.0 ± 0.14 and 9.8 ± 0.19 g/dL. Percentage of sickle hemoglobin were 0, 72 ± 1.6 and 32 ± 1.9, respectively. Oxygen saturation level (SpO_2_) in each group was 97.9 ± 0.3, 96.6 ± 0.7 and 96.8 ± 1.1, respectively. For CTF subjects, measurements were obtained before their routine blood transfusion.

Written informed consent/assent was obtained before participation in the study. All subjects completed a physical examination and history to evaluate for wellness prior to the exam. A data safety monitoring plan included a pre and post-test questionnaire to evaluate for the presence of pain and sickle cell related vaso-occlusive symptoms. The post-test questionnaire was performed immediately following the study and 24–72 h later. All subjects tolerated CFS and none of the subjects reported symptoms at either time point.

### Protocols

The experimental protocol was designed to measure the physiological responses to the CFS. The subject rested comfortably in recumbent position with their torso elevated at a 30-degree angle. The subject was allowed to rest quietly for 10 min to allow the physiological measurements to stabilize. An ice pack was then placed on the subject’s forehead for 60 sec. After the removal of the ice pack, the subject was again allowed to rest quietly for 10 min.

Respiration and electrocardiogram (ECG) were recorded continuously using Lifeshirt physiologic monitoring system (VivoMetrics, Ventura, CA). Continuous blood pressure (BP) was monitored noninvasively using Nexfin (BMEYE, Ansterdam, The Netherlands). Peripheral arterial tonometry (WatchPAT-100, Itamar Medical, Caesarea, Israel) was employed for assessment of pulse wave amplitude at the level of the finger. “PAT” noninvasively obtains a beat-to-beat plethysmographic recording of the finger arterial pulse wave amplitude. During vasoconstriction when peripheral vascular resistance increases, the pulse amplitude of PAT (PATamp) decreases (Schnall et al. 1999; Grote et al. 2003). Therefore, we used the PAT signal to detect peripheral vasoconstriction, and assumed PATamp to be inversely related to peripheral vascular resistance. Respiration, ECG and continuous BP were recorded at 200 Hz; PAT was recorded at 40 Hz.

### Data preprocessing

The beat-to-beat interval of the R-to-R wave on the ECG (RRI) and tidal volume (*V*_T_) were derived from the ECG and respiration recordings using VivoLogic software (VivoMetrics, Ventura, CA). Beat-to-beat systolic and diastolic blood pressure (SBP and DBP) were extracted from the peak and nadir of each beat on the continuous BP recording. Beat-to-beat mean arterial pressure (MAP) was approximated as a sum of 1/3 of SBP and 2/3 of DBP (Levy et al. 2007). Beat-to-beat PATamp was defined as the difference between the peak and nadir of each beat on the PAT recording. Because the PAT signal is given in arbitrary units, the beat-to-beat values of PATamp were normalized by the mean and standard deviation (STD) of the PATamp time-series during baseline (1–5 min of quiet data recording before the CFS) to allow comparison across subjects. Normalized PATamp (PATampN) was computed as follows:

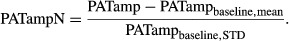
1

The above equation yields a measure of peripheral vasoconstriction/vasodilation relative to the baseline PAT amplitude in each subject, similar to a *z*-score.

All beat-to-beat signals were interpolated and then uniformly sampled at 2 Hz. The *V*_T_ signal was also down-sampled to 2 Hz. R-R interval, *V*_T_, SBP, MAP, and PATampN were aligned such that time 0 indicates the onset of the CFS. All signals were subjected to 5th-order polynomial trend removal such that each signal reflected fluctuations around zero. Further, a lowpass filter using Kaiser window (Oppenheim et al. 1999) with a passband of 0–0.5 Hz and a stopband at 0.85–1 Hz was applied to remove high-frequency (HF) noise.

### Modeling and parameter estimation

To determine the effect of fluctuations in *V*_T_ and BP on changes in RRI and peripheral resistance, reflected by PATampN, in response to CFS, we employed two linear time-varying models whose structures were similar to that published by our group previously (Chaicharn et al. 2009). For both models, any effects of autonomic activity on RRI and PATampN were assumed to have been reflected in *V*_T_ and BP fluctuations. The first model was the model of HRV. Fluctuations in RRI (ΔRRI) were assumed to be governed by two functional autonomic mechanisms: the arterial baroreflex (ABR) and the respiratory-cardiac coupling (RCC). Arterial baroreflex relates fluctuations in SBP (ΔSBP) to ΔRRI and RCC relates fluctuations in *V*_T_ (Δ*V*_T_) to ΔRRI. *ε*_RRI_ represents extraneous influences that are not captured by the ABR and RCC. The mathematical representation of the HRV model is as follows:

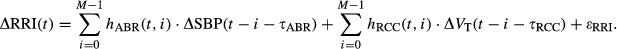
2

A second model was proposed for characterizing the key factors influencing peripheral vascular resistance variability. As peripheral resistance is modulated by the baroreflexes, we assumed a dynamic relationship between fluctuations in PATampN and fluctuations in BP. It is also well established from peroneal nerve activity measurements that respiration modulates sympathetic neural outflow (Eckberg 1995). In turn, the respiratory-related oscillations in sympathetic activity exert a strong modulatory influence on peripheral vascular tone (Malpas 2002). Previous studies have also shown that deep breaths/sighs trigger a peripheral vasoconstriction response (Bolton et al. 1936; Sangkatumvong et al. 2011). Thus, fluctuations in PATampN (ΔPATampN) were assumed to be produced through two functional mechanisms (Chalacheva and Khoo 2013): (1) baroreflex control of peripheral vascular conductance (BPC), which relates fluctuations in MAP (ΔMAP) to ΔPATampN; and (2) respiratory-peripheral vascular conductance coupling (RPC), which relates fluctuations in Δ*V*_T_ to ΔPATampN. *ε*_PATampN_ represents extraneous influences in ΔPATampN that are not captured by the BPC and RPC. The mathematical representation of the peripheral resistance variability model is as follows:

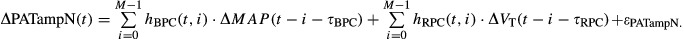
3

In equation 2 and 3, *h(t,i)* represents the time-varying impulse response of each mechanism. An impulse response quantifies the dynamics of the changes in the output resulting from a brief and abrupt unit increase in the input. For example, *h*_ABR_ quantifies the dynamics of the ΔRRI resulting from an abrupt increase in SBP of 1 mmHg. The impulse responses in this study were allowed to vary with time, thus time-varying, in order to capture changes in the system in different phases of the CFS. *M* represents the memory of the system. Based on the nature of the dynamics of interest, each impulse response was assumed to persist for 50 sampling intervals where each sampling interval was 0.5 sec (i.e., 25 sec).

It should be noted that equations 2 and 3 provide open-loop representations of the effects of BP and respiration on ΔRRI and ΔPATampN, respectively. ΔRRI and changes in peripheral resistance, in turn, are expected to subsequently affect BP, as the measurements were made with these systems operating in closed-loop mode. In order to delineate the feedback from feedforward dynamics of these closed-loop systems, it was necessary to explicitly impose causality on the models by introducing delays, *τ*, into each of the proposed mechanism. Based on the physiological considerations, *τ*_ABR_ and *τ*_RCC_ were assumed to range from 0.5 to 3 sec (Belozeroff et al. 2002, 2003) and from −3 to 0 sec (Mullen et al. 1997; Belozeroff et al. 2002), respectively. *τ*_BPC_ and *τ*_RPC_ were assumed to fall within 3.5–8 and 0–3 sec, respectively. The final estimates for each of these delays in each dataset analyzed were obtained by selecting the candidate solution that yielded the lowest criterion function value (see paragraph below).

To estimate the impulse responses, we employed a kernel expansion technique where each impulse response was represented as a weighted sum of a set of basic functions. This approach was chosen because it greatly reduces the number of parameters to be estimated and thus improves the estimation accuracy even when applied on a relatively short and noise-contaminated data segment (Marmarelis 1993). Meixner basis functions (MBF) were employed as the basis functions for the impulse responses because of their exponential decaying characteristics. In addition, they have a parameter, called the order of generalization, which allows us to have control over the rise time of the functions, making the approximation of impulse responses with slow start more accurate (Asyali and Juusola 2005). In this study, the allowed ranges of order of generalization for each mechanism were ABR: 1–5, RCC: 0–5, BPC: 2–8, and RPC: 2–8. The allowed ranges of Meixner function order (the total number of MBFs used in the expansion of an impulse response) for each mechanism were ABR: 3–6, RCC: 3–6, BPC: 2–6, and RPC: 2–6. The unknown expansion coefficients, that is the weights of the MBFs, were estimated using least-squares minimization. This least-squares minimization process was repeated for each combination of the delays, order of generalization, and Meixner function order. Minimum description length (MDL) was employed as a measure of the balance between quality of data fitting and complexity of the model (Rissanen 1982). The optimal model was selected based on the global minimum MDL.

To estimate the time-varying coefficients of the impulse responses, a recursive least-squares (RLS) algorithm was employed (Haykin 1996). Recursive least squares minimizes the weighted squared error between the model prediction and the data. In this case, the forgetting factor method was chosen, in which the weighting coefficients take the form of an exponential function where the decay rate is controlled by a parameter called the forgetting factor (*λ*). *λ* reflects the memory of the adaptive filter and can vary from 0 to 1. When *λ* = 1, all data before the present time are used in the estimation of the current parameter. Smaller values of *λ* imply that the most recent data points are weighted more heavily; however, the tradeoff is that there is greater variability in the parameter estimates as there is effectively less averaging of data. In this study, we allowed *λ* to vary between 0.85 and 0.97. The value of *λ* that minimized the squared error between the model prediction and the data was selected.

To reduce the variability in parameter estimates, we employed the following technique. Upon obtaining the initial estimate of the impulse responses using the method described above, the model prediction and the residuals (difference between data and model prediction) were calculated. Surrogate residuals were generated from the original residuals time-series by applying the Amplitude-Adjusted Fourier Transform (AAFT) technique (Theiler et al. 1992; Garde et al. 2001). This AAFT technique allowed us to reshuffle the original residuals time-series such that its Fourier Transform magnitude spectrum was preserved but the phase component was shuffled randomly. The surrogate residuals were subsequently added back to the original predicted output to generate a new output time-series. The estimation process was repeated by treating the new output time-series as if it were a new measurement of the output. The parameter estimation described above was applied to the “new” output time-series. This process was repeated 50 times – each time, the impulse response of the model was estimated. The median impulse response, computed from the 50 estimated responses, was taken to represent the final estimated impulse response.

Once the impulse response was obtained, its transfer function was computed by applying fast Fourier transform to the impulse response at each time step. Compact descriptors were then extracted from the transfer function at each time step. These descriptors were: (1) the low-frequency (LF) gain, the average transfer function magnitude between 0.04 and 0.15 Hz; (2) the HF gain, the average transfer function magnitude between 0.15 and 0.4 Hz; and (3) the overall dynamic gain, the average transfer function magnitude between 0.04 and 0.4 Hz. Based on results obtained from our previous work (Belozeroff et al. 2002, 2003; Chaicharn et al. 2009) in other applications, we focused attention on the following estimated model parameters:

- Baroreflex control of heart rate: the average gain of the ABR transfer function in the LF region (|H_ABR_|_LF_). Decreased |H_ABR_|_LF_ would be expected to reflect reduced vagal tone and/or increased sympathetic tone.
- Respiratory-cardiac coupling: the average gains of the RCC transfer function in the LF (|H_RCC_|_LF_), HF (|H_RCC_|_HF_) and overall frequency range (|H_RCC_|_Overall_) were taken to reflect vagal modulation of heart rate.
- Baroreflex control of peripheral resistance: the average gain of the BPC transfer function in the LF region (|H_BPC_|_LF_) was taken to represent sympathetic modulation of peripheral vascular conductance. However, as PATampN does not provide an absolute measure of peripheral vascular conductance, we were able only to determine the *relative* changes in |H_BPC_|_LF_ from baseline during and after CFS in each subject group.
- Respiratory-peripheral vascular conductance coupling: the average gain of the RPC transfer function in the HF region (|H_RPC_|_HF_) was taken to reflect the strength of the respiratory-vasoconstriction reflex. As in (c), since PATampN provides only a relative measure of peripheral vascular conductance, we were able only to determine how CFS may have elicited *relative* changes in |H_RPC_|_HF_ from baseline in each subject group.

### Other calculations

For each dataset, the median values of physiological measurements, namely RRI, SBP, DBP, and PATampN, were computed during baseline (1–4 min before the cold face onset) and every half-minute interval from the onset of the CFS (0 min) for 2 min. The time-varying power spectral density of RRI, SBP, and PATampN were also computed using the RLS algorithm with parameter error reduction technique described in the previous section. The spectral indices: low- and high-frequency power (denoted LFP and HFP, respectively) were extracted from the 0.04–0.15 Hz and 0.15–0.4 Hz frequency bands of the power spectral densities of RRI and SBP. For the RRI, the LF/HF ratio (LHR_RRI_) was also computed as another spectral index to reflect the “sympathovagal balance” (Task Force, 1996). Then the mean values of these spectral indices were computed during baseline and every half-minute interval from the onset of the CFS for 2 min.

### Statistical tests

Mixed model repeated measures analysis of variance (mixed model ANOVA) was applied to each of the physiological measurements, spectral indices and model-derived descriptors – LF, HF and overall dynamic gains. The model-derived descriptors were log transformed to satisfy the assumptions of ANOVA (normality and equal variance). The mixed model ANOVA tested for significant main effects for treatment group (CTL, NTF, and CTF) and time (baseline, 0–0.5, 0.5–1, 1–1.5 and 1.5–2 min), and significant interaction effects. If significant differences were detected in the main effects and/or interactions, post hoc pairwise comparisons were performed using the Tukey’s honestly significant difference test. All statistical analyses were performed using JMP statistical software, version 11.0 (SAS Institute Inc., Cary, NC).

## Results

### Physiological measurements

**Figure 1:**
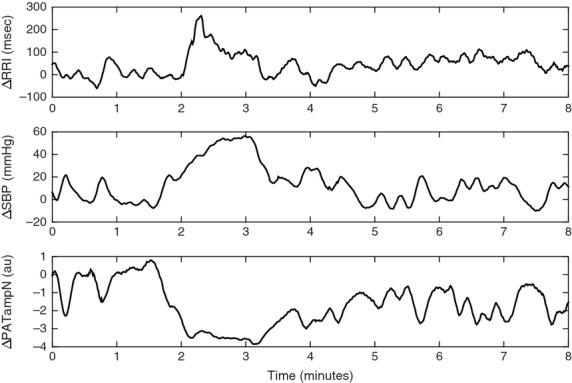
Representative tracings of changes from baseline of R-R interval (RRI), systolic blood pressure (SBP) and normalized PAT amplitude (PATampN) in a control subject before, during, and after cold face stimulation (onset at approximately 2 min).

Changes in physiological measurements, namely heart rate, BP, and peripheral resistance (represented by PATampN), in response to the CFS are summarized in Table1. Representative data of RRI, SBP, and PATampN are shown in Figure1. Only CTL subjects showed apparent increased RRI during the CFS (0–1 min). However, this increase in RRI was not enough to differentiate CTL subjects from SCD subjects. For all treatment groups, both SBP and DBP were maintained during the first half of the stimulation then increased during the second half (Table1). Post hoc comparisons showed that SBP during the second half of the CFS was overall significantly higher than baseline and 1.5–2 min after the cold face onset. Likewise, DBP during the second half of the stimulation was overall significantly higher than all time-bins being tested. The CFS caused PATampN to decrease, reflecting vasoconstriction, during and after the application of ice pack as indicated in significant time factor (*P* < 0.001). Post hoc comparisons on the time effect showed that the decreased PATampN occurred from the onset of the stimulation to 1.5 min after relative to the baseline.

**Table 1:** Changes from baseline of hemodynamic measurements during and after cold face stimulation

Variable	Group	0–0.5 min	0.5–1 min	1–1.5 min	1.5–2 min	*P*-value		
						Group	Time	Group × Time
ΔRRI (ms)	CTL	36.3 ± 29.6	31.7 ± 26.8	10.4 ± 13.3	1.3 ± 13.9	0.462	0.203	0.706
	NTF	0.7 ± 13.4	−8.3 ± 15.8	−17.9 ± 16.1	−3.8 ± 18.1			
	CTF	4.4 ± 31.9	9.4 ± 22.5	−31.9 ± 24.1	9.0 ± 23.9			
ΔSBP (mmHg)	CTL	3.5 ± 4.9	8.8 ± 6.1	6.3 ± 3.7	0.7 ± 2.9	0.366	**0.010**	0.444
	NTF	2.7 ± 4.9	13.8 ± 6.0	2.8 ± 2.8	0.6 ± 2.9			
	CTF	−4.3 ± 5.3	−0.5 ± 5.3	−3.6 ± 6.8	−3.4 ± 5.6			
ΔDBP (mmHg)	CTL	1.1 ± 2.9	3.0 ± 4.0	0.2 ± 2.5	0.2 ± 1.7	0.929	**0.004**	0.762
	NTF	1.0 ± 1.5	7.5 ± 2.6	−0.3 ± 1.9	−1.6 ± 0.9			
	CTF	−0.9 ± 3.7	3.2 ± 3.3	−0.5 ± 4.6	−0.3 ± 3.5			
ΔPATampN (au)	CTL	−2.02 ± 0.44	−2.12 ± 0.50	−1.66 ± 0.49	−0.43 ± 0.39	0.286	**<0.001**	0.463
	NTF	−1.50 ± 0.41	−1.46 ± 0.40	−1.02 ± 0.29	0.03 ± 0.35			
	CTF	−2.17 ± 0.30	−1.70 ± 0.65	−1.67 ± 0.48	−0.61 ± 0.47			

Data show mean ± standard error (SE) changes from baseline. Cold face stimulation: 0–1 min. RRI, R-R interval; SBP, systolic blood pressure; DBP, diastolic blood pressure; PATampN, normalized amplitude of peripheral arterial tonometer; CTL, healthy controls; NTF, nontransfused SCD subjects; CTF, chronically transfused SCD subjects.

*P*-value from mixed-model repeated measures ANOVA with Tukey’s honestly significant difference test for multiple comparisons. Bold values indicate statistical significance (*P* < 0.05).

### Spectral indices of cardiovascular variability

Changes in spectral indices in response to the CFS are detailed in Table2. LFP_RRI_, reflecting a combination of cardiac sympathetic and parasympathetic activity, was significantly different across subject groups (*P* = 0.015), being substantially lower in NTF compared to CTL. The same tendency was displayed by HFP_RRI_ (generally taken to reflect cardiac parasympathetic activity) but this did not achieve statistical significance. As well, both LFP_RRI_ and HFP_RRI_ in the CTF subjects tended to fall between their corresponding values in the CTL and NTF groups at each of the time-points considered. However, LHR_RRI_ (reflecting “sympathovagal balance”) displayed no significant differences among treatment groups. LFP_SBP_, which reflects BP oscillations that could be mediated sympathetically through the baroreflexes, displayed a tendency to increase during and after CFS in the CTL subjects, but this change did not attain statistical significance. There were also no significant differences in the responses among treatment groups.

**Table 2:** Spectral indices of heart-rate variability and blood pressure variability at baseline, during and after cold face stimulation

Variable	Group	Baseline	0–0.5 min	0.5–1 min	1–1.5 min	1.5–2 min	*P*-value		
							Group	Time	Group × Time
LFP_RRI_ (ms^2^)	CTL	1693 ± 358	1432 ± 210	1803 ± 522	1665 ± 325	1537 ± 353	**0.015**	0.506	0.636
	NTF	458 ± 86	604 ± 115	650 ± 161	580 ± 137	509 ± 104			
	CTF	817 ± 267	1309 ± 488	1044 ± 333	908 ± 244	1477 ± 735			
HFP_RRI_ (ms^2^)	CTL	1895 ± 766	2460 ± 1030	2660 ± 1073	2560 ± 997	2145 ± 863	0.406	0.139	0.772
	NTF	1383 ± 489	1400 ± 489	1413 ± 484	1418 ± 486	1327 ± 452			
	CTF	2948 ± 1645	1793 ± 799	2116 ± 900	1922 ± 897	1775 ± 786			
LHR_RRI_ (au)	CTL	1.54 ± 0.39	1.44 ± 0.48	1.58 ± 0.63	1.51 ± 0.55	1.53 ± 0.48	0.724	0.571	0.460
	NTF	1.08 ± 0.31	1.09 ± 0.24	1.26 ± 0.34	1.18 ± 0.33	1.10 ± 0.26			
	CTF	0.97 ± 0.41	1.12 ± 0.27	0.91 ± 0.27	1.01 ± 0.32	1.33 ± 0.41			
LFP_SBP_ (mmHg^2^)	CTL	21.1 ± 4.21	28.8 ± 7.10	32.5 ± 9.15	26.8 ± 7.69	25.1 ± 6.56	0.535	0.097	0.084
	NTF	19.6 ± 5.03	25.7 ± 6.41	25.4 ± 6.94	22.1 ± 5.85	20.1 ± 5.37			
	CTF	22.9 ± 9.37	18.6 ± 4.72	17.4 ± 7.15	21.1 ± 10.2	26.2 ± 12.0			

Data show mean ± standard error (SE). CFS: 0–1 min. LFP, low-frequency power; HFP, high-frequency power; LHR, low/high ratio; RRI, R-R interval; SBP, systolic blood pressure; CTL, healthy controls; NTF, nontransfused sickle cell disease subjects; CTF, chronically transfused sickle cell disease subjects.

*P*-value from mixed-model repeated measures ANOVA with Tukey’s honestly significant difference test for multiple comparisons. Bold value indicates statistical significance (*P* < 0.05).

### Estimated model parameters

**Figure 2:**
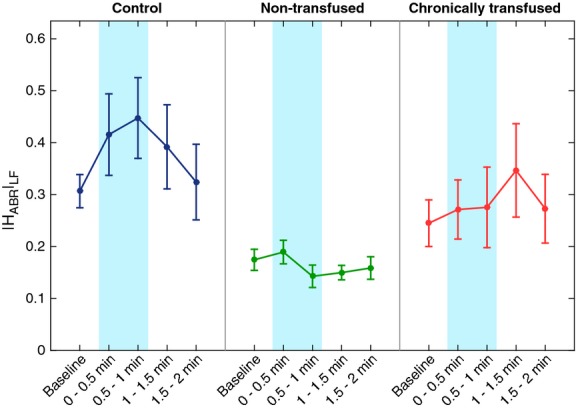
Effect of cold face stimulation (CFS) on baroreflex control of R-R interval represented by the low-frequency H_ABR_ gain (see text). Controls (CTL) displayed a transient increase in baroreflex gain during CFS with recovery toward baseline following CFS, whereas nontransfused sickle cell disease (SCD) (NTF) subjects did not show any change. Chronically transfused SCD subjects tended to display a delayed increase in gain following CFS. CTL had significantly higher baroreflex gain overall compared to NTF (*P* = 0.003).

The responses to CFS of the heart-rate baroreflex gain (|H_ABR_|_LF_) derived from the HRV model are shown in Figure2. There was a tendency for |H_ABR_|_LF_ during baseline to be highest in CTL, followed by CTF and lastly NTF subjects, but these differences were not statistically significant. However, application of the CFS accentuated these group differences, in that CFS led to an increase in |H_ABR_|_LF_ in CTL subjects but not in the NTF or the CTF subjects. In the CTF subjects, there was a small tendency for |H_ABR_|_LF_ to increase only after a delay, following removal of the CFS. The mixed model ANOVA showed a significant main effect for treatment group (*P* = 0.003). Pairwise comparisons revealed significant differences between CTL and NTF at various time-points during and after CFS application, whereas there was no significant difference between CTL and CTF.

**Figure 3:**
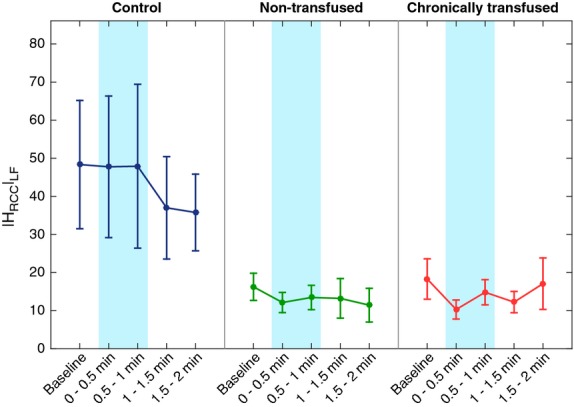
Effect of cold face stimulation (CFS) on respiratory coupling of R-R interval represented by the low-frequency H_RCC_ gain (see text). The respiratory-cardiac coupling gain did not change during CFS. Both non-transfused sickle cell disease (SCD) and chronically transfused SCD subjects showed suppressed respiratory-coupling gain compared to controls (*P* = 0.009).

Figure3 displays the effect of CFS on RCC gain, |H_RCC_|_LF_. |H_RCC_|_LF_ in CTL was higher overall compared to both SCD groups during baseline, CFS and following CFS application (*P* = 0.009). Thus, unlike the heart-rate baroreflex gain, RCC gain in CTL did not change during CFS, although it displayed a tendency (not significant) to be slightly decreased following removal of the cold face stimulus. |H_RCC_|_LF_ did not change with CFS in both NTF and CTF subjects.

**Figure 4:**
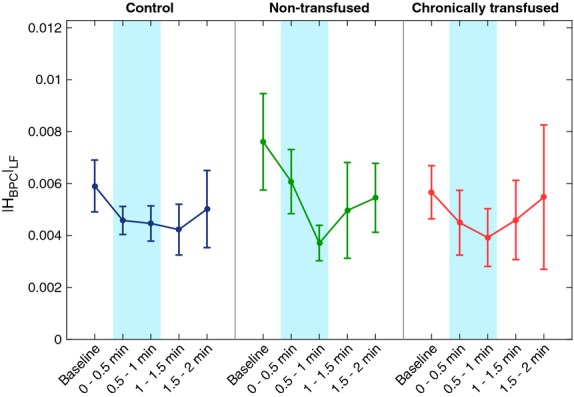
Effect of cold face stimulation (CFS) on baroreflex control of peripheral vascular conductance represented by the low-frequency H_BPC_ gain (see text). The baroreflex gain decreased from the onset of CFS until the half minute following CFS relative to baseline in all three treatment groups: controls, nontransfused sickle cell disease (SCD) (NTF) and chronically transfused SCD subjects (*P* < 0.001). NTF subjects displayed the largest drop in the baroreflex gain during CFS.

The response to CFS of the gain representing baroreflex modulation of peripheral vascular resistance (|H_BPC_|_LF_) is shown in Figure4. |H_BPC_|_LF_ in all treatment groups significantly decreased relative to baseline from the onset of CFS until the half minute following CFS (*P* < 0.001). However, pairwise comparison between baseline and the last half-minute of CFS confirmed that the NTF subjects displayed the largest drop in |H_BPC_|_LF_ during CFS, as illustrated in Figure4.

**Figure 5:**
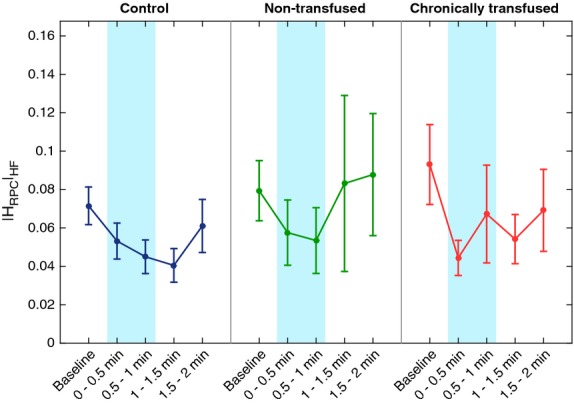
Effect of cold face stimulation (CFS) on respiratory coupling of peripheral vascular conductance represented by the high-frequency H_RPC_ gain (see text). The respiratory-vascular conductance coupling gain decreased during CFS in all three treatment groups: controls, nontransfused sickle cell disease (SCD) and chronically transfused SCD subjects (*P* < 0.001). The trend of changes over time in all treatment groups appeared to be similar.

Figure5 shows that the gain representing the respiratory-peripheral vasoconstriction reflex, |H_RPC_|_HF_, was transiently depressed by CFS in all the treatment groups (*P* < 0.001). This was the case for the LF and overall (0.04–0.4 Hz) frequency ranges as well. Post hoc comparisons showed that |H_RPC_|_HF_ during the second half of the CFS and half a minute after the stimulation were significantly lower than the baseline. The trend of changes over time in all treatment groups appeared to be similar: pairwise comparisons did not reveal any significant differences in |H_RPC_|_HF_ among treatment groups or interactions between time and treatment groups.

## Discussion

### Cold face stimulation in autonomic testing

Cold face stimulation has been employed in previous studies as a noninvasive test of autonomic function since it activates both the cardiac parasympathetic and the peripheral sympathetic nervous systems. The characteristic responses induced by the combined activation include bradycardia and peripheral vasoconstriction with subsequent increase in BP (Khurana et al. 1980; Hilz et al. 1999). Bradycardia is mediated by efferent cardiac parasympathetic pathway while peripheral vasoconstriction is mediated by efferent sympathetic pathway (Heistad et al. 1968; Hilz and Dutsch 2006). Cold face stimulation is unique in that it elicits increases in both parasympathetic and sympathetic output, whereas most other common autonomic tests, such as tilt, produce changes in parasympathetic and sympathetic activity that are opposite in direction. It was reported that subjects with a disturbance in the integrity of the trigeminal-brainstem-vagal reflex arc had absent or depressed bradycardia response to CFS (Khurana et al. 1980). Cold face stimulation is a modification of the diving reflex, which occurs with immersion of the face in water. Previous studies demonstrated that CFS could reproduce cardiac parasympathetic response with similar sensitivity to the diving reflex (Khurana et al. 1980; Hilz et al. 1999; Hilz and Dutsch 2006) and thus could be used to evaluate parasympathetic function in patients with limited ability to cooperate (Hilz et al. 1999). As such, CFS has been employed as a means to perturb the ANS in various pathological conditions such as in diabetes mellitus, brainstem stroke, Shy-Drager syndrome, familial dysautonomia, obstructive sleep apnea, or SCT/anemia (Khurana et al. 1980; Hilz et al. 1999; Chaicharn et al. 2009; Martins Wde et al. 2012).

### Model-based analysis

Using a model-based approach, we were able to detect differences in autonomic function among CTL, NTF, and CTF subjects using only noninvasive measurements of respiration, heart rate, and peripheral vascular perfusion. Cold face stimulation served the purpose of accentuating the autonomic differences across these subject groups. The model complemented the conventional spectral analyses of HRV and BP that were also applied, enabling us to detect the autonomic differences between the CTL and SCD subjects, as well as the autonomic effects of transfusion therapy on SCD subjects (Figs.3) that were not detectable in the raw data (Table1). Since the model allowed for a decomposition of the complex closed-loop mechanisms regulating heart rate and BP into simpler functional input-output components, we were able to quantify the strength of each investigated mechanism while adjusting for other contributing factors. For instance, CFS is also known to affect breathing pattern and respiratory rate, and these changes can act to confound the autonomic effects of CFS. In our model, we incorporated respiration as one of the key inputs that can affect heart rate and peripheral vascular resistance. As well, we allowed for the likelihood that application and subsequent removal of the cold stimulus would affect the parameters (e.g., gains) characterizing the model—thus, the model equations were formulated to be time-varying in the parameters. Adaptive filtering methodology was employed to track the changes in the model parameters during and after CFS (Haykin 1996). We also introduced a new technique of reducing the error in the estimates of the time-varying parameters while enabling the RLS estimation algorithm to track relatively fast changes in the system.

### Autonomic dysfunction in SCD

The present work is the first study to employ a rigorous quantitative framework for analyzing the dynamic autonomic responses of SCD subjects to CFS, and to compare the responses of those patients who have not undergone transfusion therapy (NTF) against those subjects who are undergoing transfusion therapy (CTF). Heart-rate variability, in particular LFP_RRI_, was substantially lower in NTF subjects compared to the CTL and CTF groups (Table2). These results, together with the seemingly lower HFP_RRI_ in NTF group (although not significant), imply that CTL and CTF had higher parasympathetic activity compared to NTF, suggesting that transfusion may have partially corrected the ANS defect seen in SCD.

Our inability to detect significant differences in HFP_RRI_ across treatment groups was likely due to the confounding effect of respiration. Heart-rate variability is known to be most reliable when applied under conditions of controlled breathing, whereas the CFS tended to affect the subject’s breathing pattern. To minimize the confounding effect of respiration, we used the model to estimate the RCC gain, |H_RCC_|_LF_ (Belozeroff et al. 2002, 2003; Chaicharn et al. 2009). Although in all subject groups respiratory-coupling gain did not change during CFS, |H_RCC_|_LF_ was significantly lower in both SCD groups compared with CTL (Fig.3). These findings underscore the central point that SCD subjects exhibit impaired parasympathetic modulation of heart rate. This is consistent with previous studies that found lowered parasympathetic activity in SCT subjects (Connes et al. 2006; Hedreville et al. 2008; Sangkatumvong et al. 2011). Similarly, Nebor et al. (2011) reported diminished parasympathetic activity in SCD subjects with frequent pain crises compared to healthy controls and SCD subjects who had no pain crises during the year prior to their study. Martins Wde et al. (2012) reported a smaller bradycardia response to CFS in subjects with iron deficiency anemia and SCD compared to healthy volunteers, suggesting lower parasympathetic reserves in the former groups.

The gain of the baroreflex control of heart rate was highest in CTL, followed by CTF, and lastly NTF. During CFS, baroreflex gain increased in CTL, but remained unchanged in NTF. The increase in heart-rate baroreflex gain with CFS in normals has been documented in previous studies (Hilz et al. 1999; Chaicharn et al. 2009). In CTF subjects, there was some tendency for baroreflex gain to increase but only after a delay. Taken together with the results for |H_RCC_|_LF_, these observations suggest impaired parasympathetic modulation of heart rate in NTF and CTF relative to CTL, but partial restoration of baroreflex control toward normality in the CTF group. However, the physiological mechanisms for the improved baroreflex gain following chronic transfusion therapy remain unknown at this time. Our findings are consistent with a previous study (Martins Wde et al. 2012), that reported reduction in baroreflex sensitivity in both SCD and SCT subjects, as assessed by the more invasive technique of administration of nitroglycerine and phenylephrine. Martins et al. also found that subjects with iron deficiency anemia had normal baroreflex sensitivity, suggesting that the reduction in baroreflex sensitivity in SCD was likely not the result of chronic anemia alone. On the other hand, another study reported normal baroreflex sensitivity in SCD subjects during supine and standing (Kim et al. 2009).

Although “sympathovagal balance”, as characterized by LHR_RRI_, was not different across the subject groups, the substantially lower heart-rate baroreflex gain in the SCD subjects relative to CTL suggests that the SCD subjects had elevated sympathetic tone. An inverse association between baroreflex sensitivity and sympathetic tone in a variety of conditions (e.g., hypertension, heart failure, postmyocardial infarction) has been well documented in past studies (Grassi et al. 1995; La Rovere et al. 2008). To account for this association, it has been argued that impaired baroreflex sensitivity leads to reduction in reflex sympathetic restraint, which in turns allows sympathetic overactivity to occur. In the CTL subjects of this study, application of CFS led to an increase in vagal tone (hence the bradycardia), which also increased the gain of baroreflex control of heart rate. This CFS-induced increase in heart-rate baroreflex gain did not occur in the NTF subjects; but the CTF subjects displayed a delayed, small increase.

Peripheral vasoconstriction in response to CFS occurred in all three subject groups, as evidenced by statistically significant decreases in PATampN and corresponding increases in SBP (Table1). There were no differences in the time-course of these changes in PATampN and SBP across subject groups. Reduction in PATampN was expected as CFS has been shown to induce peripheral vasoconstriction through the efferent sympathetic pathway (Khurana et al. 1980; Hilz et al. 1999). Thus, the fact that both SCD groups of subjects exhibited similar time-courses of PATampN reduction indicates that sympathetic modulation of peripheral vascular resistance remains intact in SCD. However, an important limitation of our study is that peripheral arterial tonometry does not provide an absolute measure of peripheral vascular resistance. Based on our measurements of PATampN, it was possible to only compare changes (elicited by some stimulus) relative to baseline levels. Thus, we were not able to make direct inferences about sympathetic tone in the different subject groups.

The gain corresponding to baroreflex control of peripheral vascular conductance decreased transiently following CFS in all subject groups, again indicating intact sympathetic modulation of peripheral vasoconstriction in spite of the presence of SCD (Fig.4). The time-courses for the CFS-induced reduction in baroreflex gain were largely similar between CTL and CTF subjects; however, the NTF subjects displayed reductions in |H_BPC_|_LF_ from baseline that were distinctively larger. Although the reason for this difference remains unclear at this point, we speculate that there may be an ominous clinical implication to this finding. A large reduction in the vascular resistance baroreflex gain during cold face exposure, coincident with rising BP means that there may be insufficient negative feedback from the baroreflexes to effectively counter the progressive peripheral vasoconstriction. The reduction in peripheral flow due to this transient blunting of the vascular resistance baroreflex in vulnerable SCD subjects could greatly enhance the chances for triggering local VOC and ultimately a VOC cascade.

A number of limitations in this study should be addressed. First, male and female subjects were combined for analysis of their autonomic responses to CFS because the sample size was not large enough to include gender as an independent factor although autonomic function can differ between genders. A separate analysis (not reported here) was performed to investigate the effect of gender difference. We found that there was no statistical difference in response to CFS between genders. Another limitation was the age of the recruited subjects (youngest subject was 10 years old). While age could be a strong factor affecting autonomic function, we found that exclusion of these young subjects did not change our findings in this study. Lastly, two SB0 thalassemia subjects were included in the NTF group while the rest of the SCD subjects were SS genotype. We found that the findings remained unaltered after excluding these two subjects. Thus, although differences in SCD genotype could contribute to differences in autonomic function, future studies involving larger numbers of subjects with various genotypes would be required to validate this possibility.

In summary, this study investigated the effect of CFS on SCD subjects who had and had not gone through transfusion therapy program using a time-varying modeling approach as a complement to conventional spectral analyses of heart rate and BP variability. Using the modeling approach, we found that regardless of their participation in the transfusion program, SCD subjects had reduced respiratory sinus arrhythmia, suggesting that their low parasympathetic activity is not corrected by transfusion. On the other hand, only nontransfused SCD subjects had impaired cardiac baroreflex compared to controls and chronically transfused SCD subjects, suggesting improvement in the baroreflex sensitivity following transfusion therapy. In addition, we found that the lung-peripheral vascular conductance coupling appeared to be similar in all treatment groups. However, the sensitivity of the baroreflex control of vascular resistance in nontransfused SCD subjects was distinctly lower during CFS compared to its baseline. This blunting of the baroreflex during elevated sympathetic drive (such as induced by CFS) could be a potential factor that contributes to the triggering of vaso-occlusion in SCD.
